# E-Cigarette Usage and Arthritis in the United States, a Nationwide Cross-Sectional Survey

**DOI:** 10.3389/fphar.2022.883550

**Published:** 2022-05-24

**Authors:** Yi Tian, Zhihua Jiao, Yingying Mao, Zhenyu Zhang

**Affiliations:** ^1^ Department of Rheumatology, The Second Affiliated Hospital of Dalian Medical University, Dalian, China; ^2^ Johns Hopkins School of Medicine, Baltimore, MD, United States; ^3^ Department of Epidemiology and Biostatistics, School of Public Health, Zhejiang Chinese Medical University, Hangzhou, China; ^4^ Department of Global Health School of Public Health, Peking University, Beijing, China

**Keywords:** e-cigarette, arthritis, the behavioral risk factor surveillance system, multivariable logistic regression, survey

## Abstract

**Aim:** The prevalence of the use of electronic cigarettes (e-cigarettes) has grown rapidly in the past decade in the United States. While numerous studies have demonstrated combustible cigarette is closely associated with an increased risk of arthritis diseases, little is known about the effect of e-cigarette usage on inflammatory arthritis diseases. We aimed to determinate if e-cigarette usage is associated with an increased risk of inflammatory arthritis.

**Methods:** Data were obtained from the Behavioral Risk Factor Surveillance System, which is the largest national telephone-based survey of randomly sampled adults in the United States. A total of 924,882 participants with information on e-cigarette usage and inflammatory arthritis were included. We used multivariable logistic regression to estimate the risk of arthritis associated with e-cigarette usage.

**Results:** Of the 924,882 participants, there were 30,569 (3.3%) current e-cigarette users, and 314,190 (25.9%) reported to have inflammatory arthritis diseases. In the fully adjusted model, we observed that the odds ratio (OR) (95% confidence interval) of inflammatory arthritis diseases was 1.81 (95% CI, 1.70-1.93) for current e-cigarette users compared with never e-cigarette users. The ORs of inflammatory arthritis diseases were 1.31 (95% CI, 1.18-1.47), and 1.55 (95% CI, 1.42-1.69) among sole e-cigarette and dual users compared with never e-cigarette users, respectively.

**Conclusions:** This is the first study to observe a cross-sectional association between e-cigarette usage and inflammatory arthritis diseases, and the findings were consistent in both sole-e-cigarette users and dual users. Our findings provide evidence that e-cigarette usage might be an important risk factor for arthritis diseases, which may have regulatory implications for e-cigarette control.

## Introduction

Arthritis manifests as sustained inflammation of the synovium, and uncontrolled arthritis may cause permanent joint damage, stiffness, reduced mobility, impaired functionality, and other extra-articular complications. (Scott et al., 2010). In the United States, approximately 54.4 million adults suffer from arthritis diseases, which equates to about 25% of the population, and the number is projected to be 78 million (25.9%) by 2040 (Barbour et al., 2017). Arthritis creates a profound economic burden for the person and society. In 2013, the total national arthritis-attributable medical care costs and earnings losses among adults with arthritis were $303.5 billion or 1% of the 2013 US Gross Domestic Product (Murphy et al., 2018).

E-cigarettes quickly rose in popularity in the early part of this decade, with many users viewing them as a less harmful nicotine delivery alternative to combustible cigarettes. These battery-operated devices aerosolized, commonly called “vapor” by heating a liquid mixture, which usually contains nicotine, tetrahydrocannabinol, cannabinoid oils, flavorings, and other additives. Although, at much lower levels than found in cigarette smoke, e-cigarette aerosol has been found to contain ultrafine particles and known toxins, such as acetaldehyde, acrolein, toluene, and formaldehyde (Goniewicz et al., 2014). Recently, a growing body of evidence demonstrated that e-cigarette use is associated with numerous short- and long-term deleterious health consequences, including asthma, cardiovascular, oral, brain, kidney, and pulmonary diseases (Antoniewicz et al., 2016; Martin et al., 2016; Osei et al., 2019a; Osei et al., 2019b; Huilgol et al., 2019).

Given the rapidly increasing prevalence and controversy surrounding the use of e-cigarettes in general, there is a concern about whether e-cigarettes may be associated with arthritis diseases. We used the 2016–2018 Behavioral Risk Factor Surveillance System (BRFSS), which is a nationally representative sample in the United States, to determine if e-cigarette usage is associated with an increased risk of inflammatory arthritis diseases.

## Methods

### Data Source

The Behavioral Risk Factor Surveillance System (BRFSS) is a series of cross-sectional telephone surveys conducted by the Centers for Disease Control and Prevention. Participants were selected using a disproportionate stratified sample design of the noninstitutionalized civilian population in the United States. For the current study, we combined 2016 through 2018 BRFSS data, during which e-cigarette information was obtained from participants aged 18 years or older.

### Study Population

**FIGURE 1:**
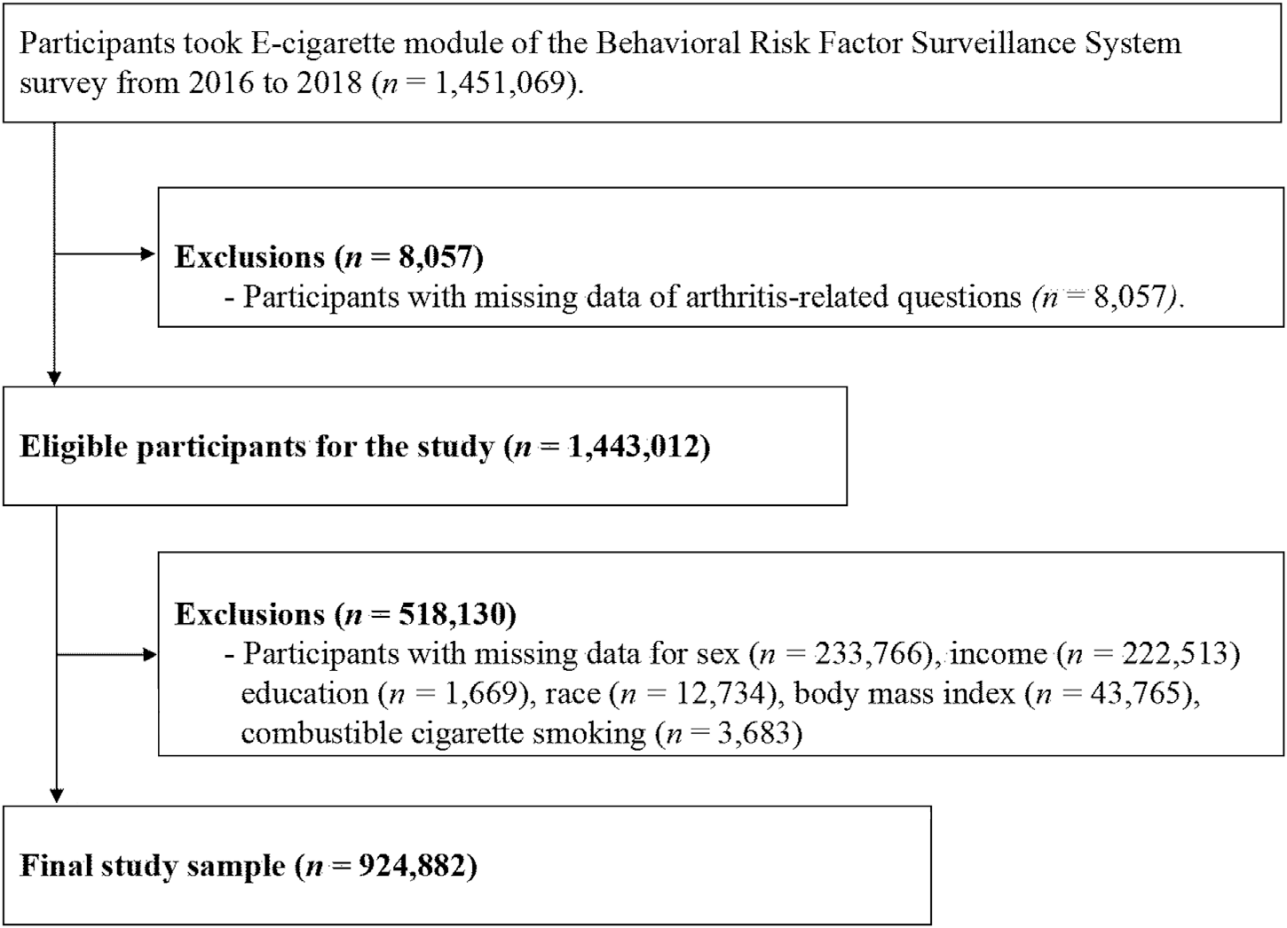
Flowchart of study participants.

This study included persons who completed the questionnaires with e-cigarette usage (*n* = 1,451,069) (Figure 1). Self-reported arthritis disease was classified based on a positive response to the question “Have you ever been told by a doctor or health professional that you have some form of arthritis, rheumatoid arthritis, gout, lupus, or fibromyalgia?” We excluded 518,130 persons with missing information on potential risk factors. The final sample included 924,882 participants.

### Definition of Arthritis Diseases

The arthritis diagnoses include sustained inflammation of the synovium, and exclude infections, tumors and trauma, including but not limited to: rheumatoid arthritis, osteoarthritis, etc. Participants who reported “yes” to the question were classified as having arthritis diseases. Participants who responded as “not sure” or who refused to answer the question were classified as missing data and were excluded from the analysis.

### Electronic Cigarette Use

Participants were first asked “Have you ever used an e-cigarette or other electronic ‘vaping’ product, even just one time, in your entire life?” Those who responded “no” were categorized as never e-cigarette users. Those who answered “yes” were categorized as ever e-cigarette users, and then asked, “Do you now use e-cigarettes or other electronic “vaping” products every day, some days, or not at all?” Ever e-cigarette users who answered “every day” or “some days” were categorized as current e-cigarette users, and those who answered “not at all” were categorized as former e-cigarette users.

Participants were asked “Have you smoked at least 100 cigarettes in your entire life?” Those who responded “no” were categorized as never combustible-cigarette smokers. Those who answered “yes” were then asked, “Do you now smoke cigarettes every day, some days, or not at all?” Those who reported “some days” or “every day” were categorized as current combustible-cigarette smokers, and those who answered “not at all” were categorized as former combustible cigarette users.

Sole e-cigarette users were defined as users who were categorized as current e-cigarette users but never combustible-cigarette users. Dual users were defined as users who categorized as both current e-cigarette users and combustible-cigarette smokers.

### Statistical Analysis

Key variables of interest included sex, race/ethnicity (white non-Hispanic, black non-Hispanic, Hispanic, other race only non-Hispanic, and multiracial non-Hispanic), age (18–24 years, 25–34 years, 35–44 years, 45–54 years, 55–64 years, and 65 + years), education levels (did not graduate high school, graduated high school, attended college or technical school, and graduated from college and technical school), annual income (<$15,000, $15,000—$25,000, $25,000—$35,000, $35,000—$50,000, and $50,000 or more), body mass index (BMI) (underweight (BMI <18.5), normal weight (18.5 ≤ BMI <25), overweight (25 ≤ BMI <30), and obese (BMI ≥30)), and combustible cigarette smoke status (never, former, and current).

We investigated potential effect modification by age, sex, race, education levels, annual income groups, and BMI. For each potential effect modifier, we evaluated effect modification by likelihood ratio tests comparing models that included an interaction (product) term between whether use e-cigarette and the effect modifier versus models without the interaction term. Stratum-specific ORs were obtained from the same interaction model by using the appropriate coefficients and variance-covariance matrix.

We performed all statistical analyses incorporating appropriate survey weights to account for the complex BRFSS sampling design and to make the estimates reported here nationally representative of the noninstitutionalized civilian population of adults aged 18 years and over in the U.S. in 2017–2018 (Rolle -Lake and Robbins, 2022). We compared the characteristics of the participants across e-cigarette use categories (never, former and current). We used logistic regression models adjusted for *a priori* potential risks to quantify the association between e-cigarette usage and the risk of arthritis diseases.

As a sensitivity analysis, we explored the possible effect of e-cigarette usage on the risk of inflammatory arthritis among young participants (age under 35 years old). All associations are presented as odds ratios (OR) with corresponding 95% confidence intervals (CI). Differences were considered statistically significant at *p* < 0.05. All analyses were conducted using Stata statistical software (version 16.0; Stata Corp LLC, College Station, Texas).

## Results

Of the 924,882 persons aged 18 years or older in our study sample in the BRFSS, 314,190 had arthritis diseases, for an overall weighted prevalence of 25.9% (95% CI, 25.7–26.1%). These data correspond to approximately 79.7 million individuals aged 18 years or older in the US population in 2010. There were 30,569 (3.3%) current e-cigarette users, and 20.2% of the e-cigarette users were 25–34 years of age. (Table 1). Compared with never e-cigarette users, current e-cigarette users were more likely to be men, with lower education levels, and lower annual income.

**TABLE 1:** Characteristics of the study participants according to e-cigarette use, BRFSS 2016 to 2018.

Variables	All Population	Never	E-cigarette Use Status	
			Former	Current
Number	924,882	775,004	119,309	30,569
Age groups, years				
18–24	44,870 (4.9)	25,766 (3.3)	14,604 (12.2)	4,500 (14.7)
25–34	95,148 (10.3)	63,384 (8.2)	25,600 (21.5)	6,164 (20.2)
35–44	111,540 (12.1)	86,336 (11.1)	20,044 (16.8)	5,160 (16.9)
45–54	151,191 (16.3)	124,681 (16.1)	21,014 (17.6)	5,496 (18.0)
55–64	208,088 (22.5)	179,220 (23.1)	22,983 (19.3)	5,885 (19.3)
65 or older	314,045 (34.0)	295,617 (38.1)	15,064 (12.6)	3,364 (11.0)
Women	495,691 (53.6)	423,872 (54.7)	57,540 (48.2)	14,279 (46.7)
Race				
White only, non-Hispanic	727,407 (78.6)	612,064 (79.0)	91,188 (76.4)	24,155 (79.0)
Black only, non-Hispanic	68,405 (7.4)	58,524 (7.6)	8,226 (6.9)	1,655 (5.4)
Hispanic	71,344 (7.7)	59,246 (7.6)	9,987 (8.4)	2,111 (6.9)
Others race only, non-Hispanic	39,717 (4.3)	32,286 (4.2)	5,925 (5.0)	1,506 (4.9)
Multiracial, non-Hispanic	18,009 (1.9)	12,884 (1.7)	3,983 (3.3)	1,142 (3.7)
Education				
Did not graduate high school	58,813 (6.4)	46,403 (6.0)	9,788 (8.2)	2,622 (8.6)
Graduated high school	246,614 (26.7)	197,018 (25.4)	38,951 (32.6)	10,645 (34.8)
Attended college or technical school	256,614 (27.7)	205,399 (26.5)	40,330 (33.8)	10,885 (35.6)
Graduated from college or technical school	362,841 (39.2)	326,184 (42.1)	30,240 (25.3)	6,417 (21.0)
Annual income				
Less than $15,000	88,798 (9.6)	67,733 (8.7)	16,578 (13.9)	4,487 (14.7)
$15,000 to less than $25,000	148,444 (16.1)	118,463 (15.3)	23,461 (19.7)	6,520 (21.3)
$25,000 to less than $35,000	98,320 (10.6)	80,637 (10.4)	14,027 (11.8)	3,656 (12.0)
$35,000 to less than $50,000	132,138 (14.3)	109,700 (14.2)	17,973 (15.1)	4,465 (14.6)
$50,000 or more	457,182 (49.4)	398,471 (51.4)	47,270 (39.6)	11,441 (37.4)
Combustible cigarette use				
Never smoker	516,871 (55.9)	486,015 (62.7)	26,952 (22.6)	3,904 (12.8)
Former smoker	269,302 (29.1)	229,861 (29.7)	29,721 (24.9)	9,720 (31.8)
Current smoker	138,709 (15.0)	59,128 (7.6)	62,636 (52.5)	16,945 (55.4)
Body mass index				
Underweight	13,955 (1.5)	10,686 (1.4)	2,435 (2.0)	834 (2.7)
Normal	283,110 (30.6)	232,505 (30.0)	40,183 (33.7)	10,422 (34.1)
Overweight	336,258 (36.4)	285,674 (36.9)	40,495 (33.9)	10,089 (33.0)
Obese	291,559 (31.5)	246,139 (31.8)	36,196 (30.3)	9,224 (30.2)

The numbers in the table are mean (SD) or count (%).

Among participants with inflammatory arthritis, the weighted prevalence of current e-cigarette and combustible cigarette users were 3.7 and 16.2%, respectively (Table 2). The weighted prevalence rates were 13.2 and 8.9% for participants without inflammatory arthritis, respectively.

**TABLE 2:** Prevalence of smoking rate of e-cigarette and combustible cigarette usage among BRFSS participants (%).

Smoking Status	Patient with Inflammatory Arthritis	Patients without Inflammatory Arthritis	Total
E-cigarette			
Never	21.1	57.5	78.5
Current	3.7	13.2	17.0
Former	1.1	3.4	4.5
Combustible cigarette			
Never	45.9	11.9	57.8
Current	16.2	8.9	25.1
Former	11.9	5.1	17.0

In the fully adjusted model, the odds ratio (OR) (95% confidence interval) of self-reported inflammatory arthritis diseases was 1.81 (95% CI, 1.70-1.93) for current e-cigarette users compared with never e-cigarette users (Table 3). The ORs of self-reported inflammatory arthritis diseases was 2.06 (95% CI, 1.93-2.20) for current e-cigarette users compared with never combustible cigarette and e-cigarette users. The ORs of self-reported inflammatory arthritis diseases were 1.31 (95% CI, 1.18-1.47), and 1.55 (95% CI, 1.42-1.69) among sole e-cigarette and dual users compared with never e-cigarette users, respectively (Table 4).

**TABLE 3:** Association between E-Cigarette use and the risk of inflammatory arthritis diseases among US adults.

E-cigarette Use Status	OR	95% CI	OR	95% CI
Never e-cigarette users	Ref (Scott et al., 2010)	—	Ref (Barbour et al., 2017)	—
Former e-cigarette users	1.48	1.43–1.54	1.69	1.62–1.75
Current e-cigarette users	1.81	1.70–1.93	2.06	1.93–2.20

Model adjusted for age group, sex, race, education levels, annual income, and BMI., ^1^Reference group were never e-cigarette users but include the combustible cigarette users. ^2^Reference group were never e-cigarette user also exclude the combustible cigarette users.

**TABLE 4:** Association between E-Cigarette use and the risk of inflammatory arthritis diseases according to combustible-cigarette smoking among US adults.

E-cigarette Use Status	Combustible Cigarette Smoking Status					
	N	Never	N	Former	N	Current
Never e-cigarette users	486,015	Ref	229,861	Ref	59,128	Ref
Former e-cigarette users	26,952	1.20 (1.10, 1.32)	29,721	1.17 (1.10, 1.25)	62,636	1.28 (1.20, 1.35)
Current e-cigarette users	3,904	1.25 (1.00, 1.57)	9,720	1.31 (1.18, 1.47)	16,945	1.55 (1.42, 1.69)

Model adjusted for age group, sex, race, education levels, annual income, and BMI.

**FIGURE 2:**
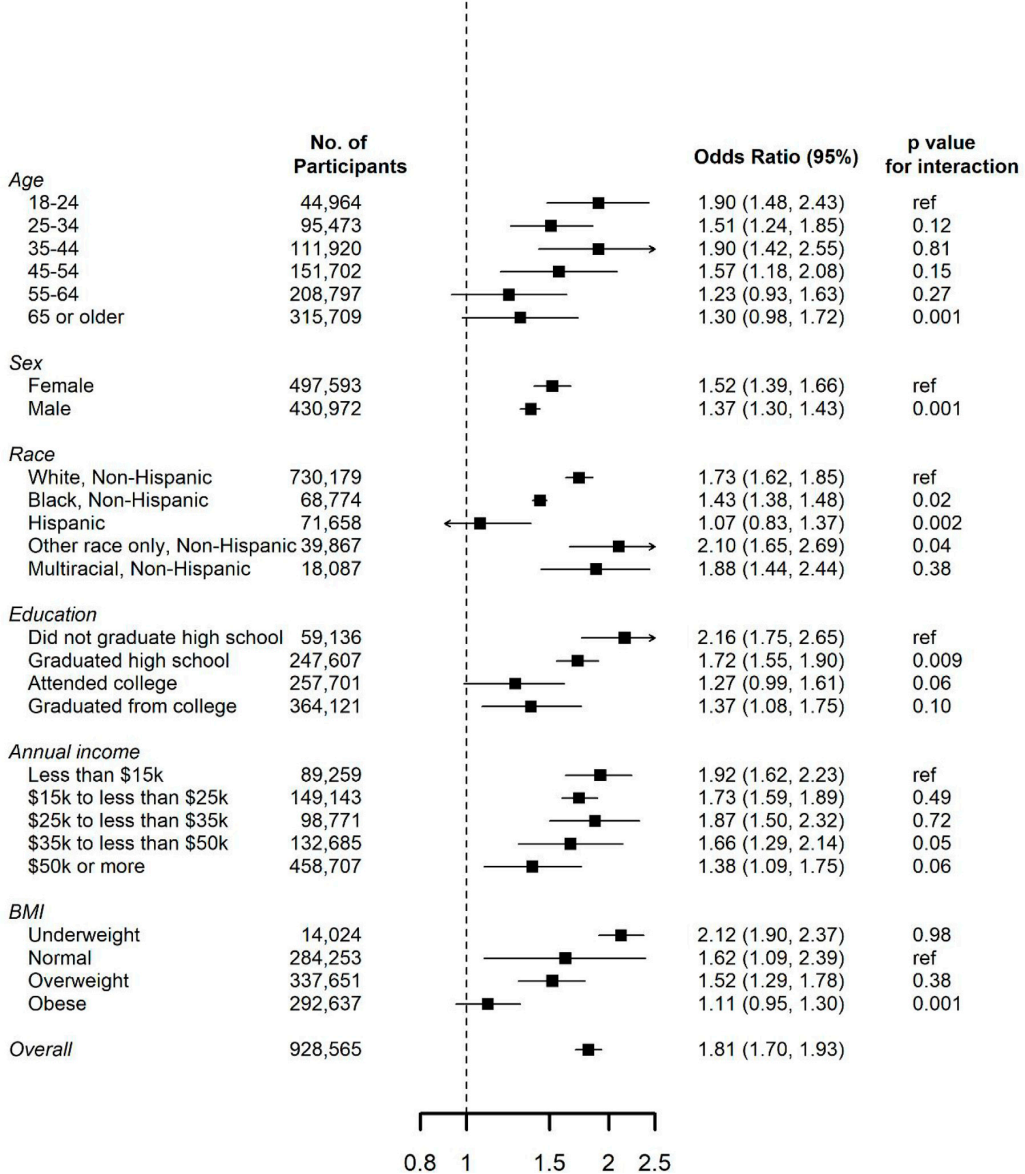
Odds ratios (95% confidence interval) for risks of inflammatory arthritis associated with current e-cigarette use in the Behavioral Risk Factor Surveillance System (BRFSS) participants, by potential risk factors. ORs were adjusted for age group, sex, race, education levels, annual income, and BMI.

In subgroup analyses, the ORs of inflammatory arthritis diseases were stronger in younger participants compared to older participants, and in males compared to females (Figure 2). In sensitivity analysis, the ORs did not results in substantially different when we restricted the analyses among participants under 35 years old (Supplementary Table S1).

## Discussion

In this representative of the U.S. adult population, we found e-cigarette usage was associated with an increased risk of inflammatory arthritis, and the results were consistent in both sole e-cigarette users and dual users. The findings from the present study have yielded several socially and clinically relevant findings.

It is noteworthy that cigarette smoking is one of the major risk factors suggested to play a crucial role in the development of arthritis due to its involvement in the pathogenesis of autoimmune diseases (Rydell et al., 2018). However, to our knowledge, no previous study has examined the impact of e-cigarette usage on inflammatory arthritis diseases. We found that current e-cigarette users have a higher risk of inflammatory arthritis compared with never e-cigarette users, especially in the current combustible cigarette group (dual users). This finding has a significant public health implication, because, initially, using e-cigarette as a potential smoking cessation tool and harm reduction strategy, the higher risks of inflammatory arthritis in dual users might indicate the undermining efforts at smoking cessation. While among the dual users, there were smokers who initiate use of e-cigarettes intending to quit may inadvertently prolong and/or sustain their nicotine addiction. Thus, exposure to an overwhelming amount of nicotine may be the potential mechanism that dual users have a high risk of arthritis in our population.

In the rapidly evolving landscape of e-cigarettes, public authorities concern that e-cigarette can become a “gateway” to smoking, teenagers may never switch to smoking but will become nicotine-dependent adults who vape, or some may initiate use of e-cigarettes in lieu of combustible cigarettes (Thorndike, 2019). In this study, sole e-cigarette users have a higher risk of inflammatory arthritis diseases, which might highlight the potential need to regulate sales and marketing of e-cigarettes to protect this vulnerable population from the onset of inflammatory arthritis diseases.

We found that current women e-cigarette smokers have a higher risk of inflammatory arthritis diseases compared to men. However, this was a subgroup analysis of our data and should be interpreted with caution. Nevertheless, female cigarette smokers face more health risks than male smokers do; they are more likely to develop lung cancer, myocardial infarction, and cognitive impairment (Risch et al., 1993; Prescott et al., 1998; Rosengren et al., 2004; Park et al., 2013). Although, current smoking was higher among men at 15.6% than women at 12.0%, the prevalence of current use of e-cigarettes was about the same for men and women in the United States (Schoenborn and Gindi, 2015; Creamer et al., 2019). Therefore, e-cigarette may be considered a risk factor inflammatory arthritis diseases for both sex, but particularly for the women who currently use of e-cigarettes.

E-cigarette use may increase inflammatory arthritis risk through gene-environment interactions, increasing inflammation and citrullination locally in pulmonary/oral mucosa or systemically, thereby inducing arthritis-related autoimmunity. Nicotine, the main constituent in e-cigarette, may have a pathogenic role in the development of arthritis diseases due to its ability to induce osteoblast apoptosis and sustained tissue hypoxia, which consequently induces osteoclast activity and bone resorption (Chaumont et al., 2018; Marinucci et al., 2018). In addition, e-cigarette use can lead to oxidative stress, altered cellular senescence, impaired host response and dysregulated repair mechanisms that can lead to arthritis diseases (Carnevale et al., 2016; Scott et al., 2018).

Although we observed e-cigarette usage was a major risk of inflammatory arthritis, our findings should be interpreted with caution because unadjusted residual confounding might remain and attenuate the association. Moreover, another caution is needed because these associations were shown epidemiologically and could not translate directly to clinical risks. Our current study has several limitations, some of which are inherent to the BRFSS data set, such as the exclusion of institutionalized individuals and lack of distinction among specific inflammatory arthritis types, including osteoarthritis, rheumatoid arthritis, gout, lupus, and fibromyalgia. As the chance of developing osteoarthritis increases with age, we restricted our analyses among young adults under 35 years old. The findings were consistent with finding from all participants. As the prevalence of e-cigarette usage was high in the youth, this finding support e-cigarette usage was a major risk of inflammatory arthritis. Additionally, the cross-sectional nature of the BRFSS precludes us from drawing firm conclusions regarding the temporality of the observed risk factor associations. Finally, the prospective cohort studies are needed to track the changes in use patterns among dual users of combustible and electronic cigarettes, which we can estimate whether combustible cigarette smokers will benefit from the substituting e-cigarettes.

In the United States, approximately 1 of every four persons aged 18 years or older estimated to have arthritis diseases. We observed that e-cigarette usage was associated with an increased risk of inflammatory arthritis diseases, with consistent results among sole e-cigarette and dual users. Given the rapidly increasing prevalence of e-cigarette usage, public policies for regulating the sale, marketing, and use of e-cigarettes, as well as relevant policies and regulations (for example, prohibit flavored e-cigarettes), are critical to improve the overall health and reduce the prevalence of arthritis diseases in the United States.

## Data Availability

The raw data supporting the conclusion of this article will be made available by the authors, without undue reservation.
