# Supplementary material for: E-Cigarette Usage and Arthritis in the United States, a Nationwide Cross-Sectional Survey
Source: Front Pharmacol. 2022 May 24;13:883550. doi: 10.3389/fphar.2022.883550 (PMC9170919; doi:10.3389/fphar.2022.883550)
Supplement: Supplementary file 1 [file Table1.docx]

**Table S1.** Association between E-Cigarette use and the risk of inflammatory arthritis diseases among US adults.

| E-cigarette use status | All respondents | Combustible cigarette smoking status | | |
| --- | --- | --- | --- | --- |
|  |  | Never Smoker | Former smoker | Current smoker |
| Never e-cigarette users | Ref | Ref | Ref | Ref |
| Former e-cigarette users | 1.43 (1.30, 1.58) | 1.06 (0.90, 1.26) | 1.04 (0.85, 1.27) | 1.09 (0.90, 1.32) |
| Current e-cigarette users | 2.13 (1.87, 2.43) | 1.23 (0.91, 1.66) | 1.46 (1.13, 1.89) | 1.60 (1.28, 2.01) |

Model adjusted for age group, sex, race, education levels, annual income, and BMI
